# Nurse Practitioner and General Practitioner Error Rates in a Large Digital Health Service: A Retrospective Cohort Analysis

**DOI:** 10.3390/nursrep14040246

**Published:** 2024-11-07

**Authors:** Louis Talay, Matt Vickers, Daisy Lu

**Affiliations:** 1 Faculty of Arts and Social Sciences, University of Sydney, Sydney, NSW 2050, Australia; 2 Eucalyptus, Sydney, NSW 2000, Australia; matt@eucalyptus.health (M.V.);

**Keywords:** nurse practitioner prescribing, prescribing errors, digital health, chronic disease, community health, safety, healthcare efficiency, general practitioner prescribing

## Abstract

Background: Nurse practitioners have been prescribing medication within a narrow scope of practice throughout the world for several decades as a means of meeting rising demand for community health services. Prominent medical bodies have alleged that the Australian government’s decision to remove the need for general practitioner collaboration in the context of a nurse practitioner prescribing medication compromises patient safety. Objectives: This study aimed to determine whether nurse practitioner prescribing increases patient risk relative to general practitioner prescribing in a large digital health service. Methods: Investigators retrospectively analyzed prescription errors from all audited consults of the Eucalyptus Australia service over a 6 month period between 1 October 2023 and 31 March 2024. Results: Of the 8359 consults, errors were observed in 911 (14.22%) of NP and 417 (21.37%) of general practitioner consults and this difference was found to be statistically significant, *X*^2^ (1, N = 8359), =57.33, *p* ≤ 0.001. No statistically significant difference was observed in the incidence of high-risk or never events between nurse practitioners and general practitioners. Most high-risk and never events pertained to medical contraindications, insufficient side-effect counselling, and the insufficient assessment of a patient’s medical history. Conclusion: These findings suggest that nurse practitioners are capable of safely performing patient assessments and prescribing medications for a select range of conditions in digital health services.

## 1. Introduction

The nurse practitioner (NP) role was created in the 1960s in the United States as a means of meeting the increasing demand for community health services [1]. A defining feature of this role, i.e., a feature that distinguishes it from that of a standard nurse, is an NP’s authority to prescribe medications [2]. Over the ensuing decades, nurse prescribing was introduced in other parts of the world, including Europe, New Zealand, Canada and the United Kingdom (UK) [2]. Throughout these regions, NPs (sometimes referred to as Nurse Specialists in European countries) obtain their prescribing authority through the completion of a master’s-level university degree and several years of clinical experience [3]. NP prescribing scope varies from country to country but is generally bound by restrictive protocols and/or formularies [3].

In Australia, NP roles were formally introduced into the national health system in 1998, with the first pieces of NP prescribing legislation coming into effect in several jurisdictions in 2001 [4]. Similar to other Anglo-Saxon countries, Australia requires nurses to complete a master’s degree and undergo 5 years of clinical experience to obtain prescribing authority [3]. Despite differences across state jurisdictions and various legislative changes, Australian NPs have been able to prescribe medications within their scope of practice since the early 2000s. In 2010, the Australia government granted NPs access to medications on the public benefits scheme (PBS), which significantly broadened their prescribing scope [2]. However, the NP PBS legislation contained a ‘collaborative arrangement’ clause, stipulating that NPs could only prescribe PBS-subsidized medications if they had a written agreement with a doctor or were employed by a service with one or more doctors [5,6]. In response to rising healthcare access issues and general practitioner (GP) shortages, the Australian government convened a Strengthening Medical Taskforce in 2022 [7]. One of the recommendations of the taskforce was to ‘better utilize NPs to deliver person-centred care’ and to ‘harness their full strengths and skills’. In response to the taskforce’s report, the Australian government removed the NP collaborative arrangement requirement for prescribing medications in early 2024 [7,8]. Prominent Australian medical bodies have publicly expressed their concern with this decision’s impact on care quality and safety [9,10].

Although the criticism from medical bodies tends to be framed in general quality and safety terms, recent commentary has directly questioned NP prescribing safety. In response to the government’s Removal of Requirement for a Collaborative Agreement Bill (RRCAB), the president of Australia’s largest GP organization argued that “GPs are the only trained medical specialists in whole-person care, from cradle to grave, which keeps Australians healthy and well” [10]. Recent systematic reviews have revealed that NP care delivers comparable health and safety outcomes to GP care across a number of clinical markers in primary care settings [11,12]. In some markers, such as blood pressure outcomes and patient satisfaction, NP care has even been found to be superior to GP care [11]. However, none of these findings have been taken from Australian care settings. Australian research on NP prescribing appears to be limited to regulatory confusion and constraints [13], liability concerns [14], employee characteristics [15], and cost-benefit analyses [16]. In 2023, a study also assessed Australian NP attitudes toward the idea of expanding prescribing authority to registered nurses in response to relevant advocacy from the Nursing and Midwifery Board of Australia and International Council of Nursing [17]. A key takeaway from that study was that most NPs recognized the importance of their master’s degree qualification regarding prescribing safety. In summary, while international reviews have demonstrated NP and GP prescribing safety to be comparable, no such research has been conducted in Australian care settings.

In acknowledging the scarcity of data on Australian NP prescribing, Fong et al. emphasized two related points [18]. Firstly, they noted that “NPs tend to prescribe in differing contexts of practice to provide care in underserved populations”. And secondly, they stressed the need for future research on NP practice in these contexts to better understand NP prescribing capacity and inform policy [2]. Arguably the largest ‘differing’ context in modern NP prescribing is digital care. A recent publication from the Australian Digital Health Agency reported that over 189 million electronic prescriptions had been issued between May 2020 and January 2024 from GPs and NPs [19]. The rising demand for digital care coupled with unmanageable GP workloads is likely to result in an increased need for NP prescribing in the digital care space [20,21]. Research has demonstrated the comparable safety of digital and face-to-care (F2F) care across multiple conditions, interventions, and metrics [22,23,24]. At present, the most salient utility of digital modalities appears to be in chronic care management, whereby clinicians need to monitor patients’ conditions regularly without having to physically touch them (unless they present with abnormalities) [23]. A key benefit of digital modalities in all healthcare contexts, according to an Australian Government study, is their facilitation of data management and clinical decision support tools [22]. However, to the knowledge of the authors, scholars are yet to investigate NP prescriber safety in digital care settings.

Eucalyptus is one of Australia’s largest digital health providers, having treated over 600,000 patients since launching in 2019 [25]. The service provides treatment for a select number of conditions, including overweight and obesity, fertility issues, dermatological conditions, and sexual health disorders, and has expanded its operations to Germany, Japan and the UK. All care has been hitherto delivered asynchronously via an online platform and mobile phone app. Eucalyptus patient assessments and prescriptions were performed exclusively by GPs until July 2023 when a significant number of NPs began performing these tasks. This study aims to determine whether NP prescribing increased patient risk relative to GP prescribing across all Eucalyptus care services over a 6 month period. It is believed that this investigation will generate vital preliminary findings on NP prescribing safety in digital care settings and in the Australian healthcare system in general.

## 2. Materials and Methods

### 2.1. Study Design

The study retrospectively analyzed prescribing error rates at the Eucalyptus digital health service over a 6 month period between 1 October 2023 and 31 March 2024. All study data were retrieved from the Eucalyptus clinical auditing team’s issue tracking repository on Jira (Version 9.4, Atlassian, Sydney, Australia)—a project management tool. The investigation’s ethics were approved by the Bellberry Human Ethics Committee on 22 November 2023 (No. 2023-05-563-A-1). The study followed the Strengthening the Reporting of Observational Studies in Epidemiology Statement (STROBE) guidelines. It also conformed to the “Statement on Human Experimentation” by the National Health and Medical Research Council of Australia, with all Eucalyptus practitioners who were employed over the study period consenting to their anonymized consults being used in peer-reviewed research.

### 2.2. Eucalyptus Prescribing and Clinical Auditing Processes

Prospective Eucalyptus patients complete a pre-consultation questionnaire relevant to the condition they are seeking treatment for. A GP or NP will review the patient’s responses and often request further information, such as test results and photos, to determine patient eligibility for the relevant service. During the 6 month study period, all Eucalyptus services included prescription medication, i.e., all patients were prescribed medication relevant to their condition. Once a GP or NP decides on the appropriate medication, they forward the script to one of the company’s partner pharmacy networks (Cloud, YSCP, IWG, or Evermed), who determine the exact dispensing pharmacy according to supply and patient location. All patient data, including their communication with their NP or GP and the latter’s prescribing decision, are automatically uploaded to a patient’s profile in the Eucalyptus issue tracking repository in Jira.

The repository uses analytical tools to identify prescribing errors, ranging from drug–drug interactions to ineligible demographic profile to the failure to conduct counselling. Prescriber decisions that the repository’s algorithm recognizes as high-risk errors or ‘never events’ are automatically uploaded to thematic dashboards that the Eucalyptus clinical auditing team reviews every 24 h. In addition to this protocol for high-risk events, the team performs 3 other types of manual audits: ad hoc audits; random audits; and new prescriber audits. Ad hoc audits are performed whenever the Eucalyptus clinical auditing team receives internal or external insights about potential misprescription. Random audits are conducted at a frequency consistent with a 95 percent confidence interval of the total volume of Eucalyptus patients, excluding patients who have already had their prescriptions audited. Finally, new prescriber audits are performed for the first 100 consultations of any new Eucalyptus GP or NP prescriber, or all consultations during the prescriber’s first week of practice (if this number exceeds 100). All misprescription errors are assigned a severity rating between 1, ‘low severity’ and 4, ‘never event’. Descriptions and examples of each error rating are presented in Table 1. It is important to note that these ratings are based on ‘potential harm’, i.e., worst-case scenarios, in order to remind Eucalyptus auditors and prescribing clinicians of the importance of timely intervention. Thus, the descriptions of ‘never’ and ‘high-risk’ events in the severity matrix arguably appear more serious than the reported misprescription errors. To limit any potential practitioner assessment bias, Eucalyptus clinical auditors are unable to see the practitioner type in any of the 4 auditing methods.

### 2.3. Data Collection

Investigators retrieved all data from the Eucalyptus clinical auditing repository on Jira. This data included all misprescription errors, along with the total number of consults and consult audits for all Eucalyptus digital health services over the 6 month study period. The full list of services is as follows: overweight and obesity, erectile dysfunction, premature ejaculation, female fertility, contraception, acne, hyperpigmentation, skin ageing, and hair loss. Although Eucalyptus auditors can identify patients from consult records by accessing a separate patient profile link (for emergency purposes), none of the investigators had this authority and therefore all data used in the study were deidentified.

### 2.4. Data Analysis

To achieve the study outcome of comparing NP and GP prescription error rates in the Eucalyptus digital health service, data were organized into both severity (harm Scores 1–4) and the practitioner type (GP or NP) categories. As both error status (yes or no) and practitioner type represent categorical variables, chi-square tests were used to determine whether error rates between practitioners were statistically significant. In the error column of the chi-square contingency tables, the ‘no’ cell represented the total number of audits during the study period in which errors for the given practitioner type were not detected. Following this, the denominator used in NP and GP error rate calculations was the total number of audits conducted on each the respective clinician groups. Chi-square tests were run across all 4 severity levels.

## 3. Results

Between 1 October 2023 and 31 March 2024, 8359 quality and safety audits were conducted on Eucalyptus consults, including 6408 NP consults and 1951 GP consults. From these audits, a total of 1328 prescriber errors were detected. A total of 911 errors came from NP consults and 417 from GP consults, representing cohort error rates of 14.22% and 21.37%, respectively. Errors were then broken down into the four severity categories (Table 2). Of the 911 NP errors, 17 (0.27% of NP consult audits) were recorded by auditors as never events, 100 (1.56%) as high-risk events, 447 (6.98%) as medium-risk events, and 347 (5.42%) as low-risk events. Of the 417 errors detected in GP consults, 3 (0.15% of GP consult audits) were recorded as never events, 28 as high-risk events (1.44%), 236 as medium-risk events (12.10%), and 150 (7.69%) as low-risk events.

Chi square tests were conducted across all severity levels (Table 2). The difference between NP and GP misprescription rates was not statistically significant for never or high-risk events. However, the analysis found that the higher error rates observed among GPs relative to NPs for medium- and low-severity events were statistically significant, at *X*^2^ (1, N = 8359) = 52.27, *p* ≤ 0.001 (medium-risk events) and *X*^2^ (1, N = 8359) = 13.82, *p* ≤ 0.001 (low-risk events). To create larger subgroups, severity ratings were merged into a binary: combined high-risk and ‘never’ events and combined medium- and low-risk events. Combined medium and low-risk error rates were statistically higher among GPs, *X*^2^ (1, N = 8359) = 67.44, *p* ≤ 0.001. When all severity levels were combined, i.e., when looking at all errors together, a chi-square test found that the higher rate observed among GPs (21.37%) relative to NPs (14.22%) was statistically significant, *X*^2^ (1, N = 8359) = 57.33, *p* ≤ 0.001.

Never events were evenly distributed among the four conditions treated at Eucalyptus (Table 3). A total of seven were observed in sexual health prescriptions, six in fertility prescriptions, four in weight-loss prescriptions, and three in dermatological prescriptions. Whereas never events were only detected in GP consultations for weight-loss and sexual health, NP never events were found across all four conditions. Medical contraindications accounted for three-quarters of the total number of never events (15/20).

The vast majority of high-risk errors occurred in sexual health (56.25%) and weight-loss (38.28%) consultations (Table 4). Errors were distributed across all five error types in the case of the weight-loss category and four of the five error types in the sexual health category. Whereas GP errors were spread relatively evenly across condition and error types, a disproportionately high number of NP high-risk errors pertained to insufficient side-effect counselling for sexual health consultations (31%) and an insufficient assessment of patient medical history in weight-loss consultations (20%).

## 4. Discussion

To our knowledge, this is the first study to have compared NP and GP error rates in an Australian healthcare setting. It is well established that NP prescribing represents a key means of meeting the increasing demand for community health services. Although NPs have been prescribing medication within their scope of practice in Australia (and other countries) for several decades [2,3], the safety of NP prescribing has been seriously questioned since the 2024 RRCAB [9,10]. A previous literature review had emphasized a need for assessing Australian NP prescriber safety in contexts where the practice’s utility is maximized, such as in services for underserved populations [18]. Eucalyptus Australia does not comply with this latter description as it is a relatively expensive service that primarily serves people of Caucasian ethnicity. However, it is one of Australia’s largest digital health services and arguably represents the modality that holds the most potential utility for NP prescribing and the expansion of care access. Following this, the study also appears to be the first quantitative analysis of NP prescriber safety in a digital care service.

The analysis found that overall, NP prescribing was as safe as GP prescribing over a 6 month period in the Eucalyptus Australia digital health service. Of the 8359 Eucalyptus consults that were audited, error rates of 14.22% and 21.37% were observed among NPs and GPs, respectively. Although it may be tempting to argue that NP prescribing was safer than GP prescribing given the above difference was found to be statistically significant, an analysis of severity levels revealed that the difference stemmed from low- to medium-risk events, which as the rating descriptions (Table 1) indicate, are not likely to result in significant patient harm. Another interesting finding was that high-risk or never events were detected in over 1.5% of NP and GP consults. This figure appears a little high, even despite the unavailability of high-risk error rates in comparable services.

These findings have several potential implications for the Australian healthcare system and digital care services in general. Firstly, they suggest that NPs can prescribe medications with a comparable degree of safety to GPs across a range of conditions in digital settings. Increased NP prescribing for weight-loss and sexual health treatment in particular could generate significant efficiency gains for the Australian healthcare system, given the rising obesity and sexual dysfunction rates throughout the Western World [26,27]. Secondly, they may be interpreted as preliminary evidence that digital modalities can play a major role in mitigating GP workload issues and improving access to chronic care. Further research should aim to assess NP prescribing safety in other digital services and for other chronic conditions such as diabetes and mental health disorders. Finally, the discovery that over 1.5% of NP and GP consults contained high-risk or never events highlights the need for dedicated analyses of the clinical governance protocols of digital services like Eucalyptus. The Australian Government has acknowledged that digital modalities facilitate data management and clinical decision support tools [22]. However, detailed analyses of how these tools are used in real-world services will contribute to their optimization and, feasibly, lower prescribing error rates. Further research of this nature could also lead to the establishment of national digital prescribing standards.

This study has multiple limitations. Firstly, it only analyzed NP and GP prescription errors over a 6 month period. Secondly, the Eucalyptus Australia service only provides treatment for a select number of conditions and although these conditions can all be described as chronic, the findings of this study cannot be extrapolated to the broader chronic care context as each condition comes with unique treatment criteria. Thirdly, the analysis only included Eucalyptus consults that were reviewed by the service’s clinical auditing team, which accounted for 6.59% of the 127,258 consults that were conducted over the 6 month study period. While the team’s auditing process is comprehensive, combining four discrete methods, one of which utilizes algorithms to automatically detect high-risk and never-events, it is possible that some low- and medium-risk events were missed. And finally, all prescription reviews were conducted by employees of the Eucalyptus service whose decisions may have been influenced by a range of unconscious biases towards the company. Any conclusion drawn from this study needs to be tempered with an acknowledgement of these limitations.

## 5. Conclusions

The study found that NP prescribing was as safe as GP prescribing over a 6 month period in the Eucalyptus Australia digital health service. This suggests that NPs are capable of safely performing patient assessments and prescribing medications for a select range of conditions in digital health services. Given the inability of the overburdened Australian GP workforce to meet the increasing demand for chronic care services, this finding could be of interest to health policymakers. Although research on other digital services and treatment types is needed to draw stronger conclusions about NP prescribing safety, this study lays an important foundation for such investigations. The study also adds vital nuances to the emerging literature on digital chronic care services by highlighting the potential of NP prescribing in these settings and the conditions NPs can safely prescribe for. Results from this study will hopefully encourage comparable investigations of other NP prescribing services and the development of national safety standards for digital prescribing.

## Figures and Tables

**Table 1:** Eucalyptus prescribing error severity ratings.

Severity Rating	Description	Example
4-Never event	Death or likely permanent harm which is not reasonably expected as an outcome of healthcare treatment	Patient prescribed Liraglutide with a known history of pancreatitis.
3-High	Temporary major harm or permanent consequences which are not reasonably expected as an outcome of healthcare treatment	Patient with congestive cardiac failure prescribed minoxidil without being counselled by NP or GP.
2-Medium	Minimal/minor harm which is not reasonably expected as an outcome of healthcare treatment	Patient indicates relatively high blood pressure in initial questionnaire and is prescribed erectile dysfunction medication without NP or GP requesting additional blood pressure assessment.
1-Low	Narrowly avoided harm	Patient prescribed contraception medications but clinician does not confirm they have counselled them on possible side effects.

**Table 2:** Results from chi-square analyses.

	NP		GP				
	N	(%)	N	(%)	*X* ^2^	*p*-Value	*Phi*
Never events					0.78	0.377	0.010
Observed	17	0.27	3	0.15			
Not observed	6391	99.73	1948	99.85			
High-risk events					0.01	0.930	0.001
Observed	100	1.56	28	1.44			
Not observed	6308	98.44	1920	98.41			
Medium-risk events					52.27	<0.001 ***	0.079
Observed	447	6.98	236	12.10			
Not observed	5961	93.02	1715	87.90			
Low-risk events					13.82	<0.001 ***	0.041
Observed	347	5.42	150	7.69			
Not observed	6061	94.58	1801	92.31			
Combined never and high-risk events					0.05	0.82	0.002
Observed	117	1.83	31	1.59			
Not observed	6921	98.60	1920	99.52			
Combined medium and low-risk events					67.44	<0.001 ***	0.090
Observed	794	12.40	386	19.78			
Not observed	5614	87.60	1565	80.22			
All errors					57.33	<0.001 ***	0.083
Observed	911	14.22	417	21.37			
Not observed	5497	85.78	1534	78.63			

*** *p* < 0.001.

**Table 3:** Never events by condition, type and practitioner.

Condition	Error Type	NP	GP	Example/Description
Weight				
	Medical contraindication	1	1	Patient with history of pancreatitis was prescribed Semaglutide
	Insufficient assessment of medical history	1	1	Patient indicated a history of gallstones, but GP did not clarify whether they were removed before prescribing
Sexual health				
	Medical contraindication	5		Patient was prescribed Tadalafil and is currently taking a supplement that contains nitric oxide
	Insufficient side-effect counselling	1		NP failed to confirm whether patient was taking antidepressants and did not provide counselling on Tadalafil
	Insufficient assessment of medical history		1	Patient indicated they were taking antidepressants, but GP did not seek further information before prescribing.
Skin				
	Medical contraindication	3		Patient is pregnant and was prescribed Tretinoin.
Fertility				
	Medical contraindication	6		Patient was prescribed Levlen despite having a body mass index (BMI) significantly higher than the medication’s recommended cut-off point

**Table 4:** High-risk errors by condition, type and practitioner.

Condition	Error Type	NP	GP	Example/Description
Weight				
	Medical contraindication	6	6	Patient indicated they were trying to conceive but was still prescribed Semaglutide
	Ineligible demographic profile	3	1	Patient was prescribed Liraglutide despite being 0.4 kg/m^2^ below the medication’s recommended BMI cutoff
	Incorrect medication or dose		2	Patient prescribed 1 mg of Semaglutide as initial dose
	Insufficient assessment of medical history	20	1	Patient indicated they regularly engage in binge eating, but NP prescribed Semaglutide without further investigating
	Insufficient side-effect counselling	9		Patient indicated they had anxiety but was not counselled about possible worsening of mood.
Sexual health				
	Medical contraindication	13	2	Patient prescribed paroxetine despite indicating they were taking Ritalin
	Incorrect medication or dose	2	1	NP told patient they were ineligible for paroxetine but still prescribed them this medication.
	Insufficient assessment of medical history	12	1	Patient indicated their mood was low during initial quiz but GP did not ask further questions.
	Insufficient side-effect counselling	31	5	Patient indicated they were using recreational drugs but were not advised of potential side effects/interactions with Paroxetine
Skin				
	Medical contraindication	2	4	Patient indicated they were trying to conceive and was prescribed Hydroquinone, Tretinoin, Hydrocortisone and Kojic acid.
	Insufficient side-effect counselling	1	2	Patient was breastfeeding and was prescribed Tretinoin without any counselling on proper handwashing
Fertility				
	Insufficient assessment of medical history	1	3	Patient had a body mass index of 48.22 kg/m^2^ and was prescribed Levlen

## Data Availability

The data presented in this study are available from the corresponding author on reasonable request.

## Public Involvement Statement

There was no public involvement in any aspect of this research.

## Guidelines and Standards Statement

This manuscript was drafted against the Strengthening the Reporting of Observational Studies in Epidemiology (STROBE) Statement: guidelines for reporting observational studies.
